# Humpback whale migrations to Antarctic summer foraging grounds through the southwest Pacific Ocean

**DOI:** 10.1038/s41598-018-30748-4

**Published:** 2018-08-17

**Authors:** V. Andrews-Goff, S. Bestley, N. J. Gales, S. M. Laverick, D. Paton, A. M. Polanowski, N. T. Schmitt, M. C. Double

**Affiliations:** 1 Australian Marine Mammal Centre, Australian Antarctic Division, 203 Channel Highway, Kingston, Tasmania 7050 Australia; 2 Institute for Marine and Antarctic Studies, Private Bag 129, University of Tasmania, 7001 Tasmania, Australia; 3 Blue Planet Marine, PO Box 919 Jamison Centre, 2614 Canberra, ACT Australia; 4 Australian Antarctic Division, 203 Channel Highway, Kingston, Tasmania 7050 Australia

## Abstract

Humpback whale (*Megaptera novaeangliae*) populations typically undertake seasonal migrations, spending winters in low latitude breeding grounds and summers foraging in high latitude feeding grounds. Until recently, a broad scale understanding of whale movement has been derived from whaling records, Discovery marks, photo identification and genetic analyses. However, with advances in satellite tagging technology and concurrent development of analytical methodologies we can now detail finer scale humpback whale movement, infer behavioural context and examine how these animals interact with their physical environment. Here we describe the temporal and spatial characteristics of migration along the east Australian seaboard and into the Southern Ocean by 30 humpback whales satellite tagged over three consecutive austral summers. We characterise the putative Antarctic feeding grounds and identify supplemental foraging within temperate, migratory corridors. We demonstrate that Antarctic foraging habitat is associated with the marginal ice zone, with key predictors of inferred foraging behaviour including distance from the ice edge, ice melt rate and variability in ice concentration two months prior to arrival. We discuss the highly variable ice season within the putative foraging habitat and the implications that this and other environmental factors may have on the continued strong recovery of this humpback whale population.

## Introduction

Migration is a large-scale class of animal movement driven by resource quality or availability (e.g., breeding habitat, seasonal food resources)^1^. Perhaps the most classic concept of migration is the long-distance movements of many birds and mammals characterised by breeding at one end of the migratory pathway and feeding at the other. Most humpback whale (*Megaptera novaeangliae*) populations undertake seasonal migration, spending the winter in low latitude breeding grounds and the summer foraging in high latitude and productive feeding grounds^2^. Historically, our understanding of whale migration has been informed via examination of whaling records and Discovery mark recoveries (a metal tube stamped with a unique serial number, that was fired into the whale, remaining embedded until the whale was captured and flensed^3^), which provide a coarse description of the spatial and temporal characteristics of movement. More recently, non-lethal methods such as photo identification (for example^4^) and genetic analyses (for example^5^) have provided similar point location data. These data, however, provide no detail on the movements in between mark and recapture.

Achieving a more detailed understanding of movement has been difficult for marine mammals because they are highly mobile, diving animals that spend relatively little time at the surface^6^. Whales are no exception with the majority of movement occurring in remote, often inhospitable, areas with no survey effort^7^. The development of satellite tagging technologies has provided an extremely valuable, non-lethal technique to collect high-resolution movement data over biologically relevant time scales^1^. For example, satellite tags recently revealed novel migratory pathways of New Caledonia humpback whales that utilise seamount habitats both during the breeding season and while migrating^8^. Combining historical whaling and sightings data, Branch *et al*.^9^ hypothesised that pygmy blue whales (*Balaenoptera musculus brevicauda*) migrate between Australia and Indonesia along Australia’s western coastline – a migratory path which has since been confirmed using satellite telemetry^10^. In these and other cases (for example^11^), satellite telemetry provided new detailed movement information and identified habitat important to the conservation and management of whale species.

Improvements in tagging technology, particularly advances in sensor, storage and transmission capabilities, have brought improvements in the amount and quality of data received^12,13^. Concurrently, statistical techniques have evolved and analytical methods such as state space modelling (SSM) are increasingly being applied to tag based movement data^14–16^. SSM methods combine a process model (for animal movement) with an observation model (for the tracking data), to provide an estimation of the unobserved behavioural state of the animal^17^. This approach enables inference about the nature of that behaviour – for example, two-state models commonly differentiate more localised search (foraging, resting) behaviour from more directed transiting behaviour (migration^18^). These inferred behaviours can be examined relative to the animal’s biophysical environment, enabling telemetry data to provide an understanding of the ecological factors influencing animal movement and habitat selection (e.g.^19^). The influence of directly measured and/or remotely sensed factors that may generate or concentrate resources can therefore be explored; for example environmental variables such as sea ice concentration^20^, bathymetric depth and gradient^21^, chlorophyll*-a* concentration^22^ and sea surface temperature^21,23^ have all been demonstrated to influence the “search” behaviour of cetaceans.

Obtaining a detailed understanding of the movement of wide ranging species such as humpback whales is important for informing management policy in the face of environmental variability and long term change associated with anthropogenic forcing^14,24^. The east Australian humpback whale population, designated as breeding stock E1 by the International Whaling Commission (IWC), migrates along the east coast of Australia and was hunted to near extinction in the 1950s and early 1960s^25,26^. However this population is now considered 58–98% recovered at a population size of 24,545 whales (95% CI 21,631–27,851) with no evidence that the observed exponential rate of growth is slowing down^27^. Over multiple years (2008–2010) we deployed satellite tags on east Australian humpback whales near both the feeding and breeding grounds to collect the first high resolution movement data for this population. We aimed to describe migratory movement and identify foraging habitat. Using a SSM to infer foraging behaviour we investigate one factor that we hypothesise supports this population’s sustained recovery: access to productive feeding grounds. Here we identify the environmental factors that characterise the key Antarctic foraging habitat, discuss usage of ice associated habitats in the face of change and the role of supplemental feeding in temperate grounds.

## Results

### Whale movement

**Figure 1:**
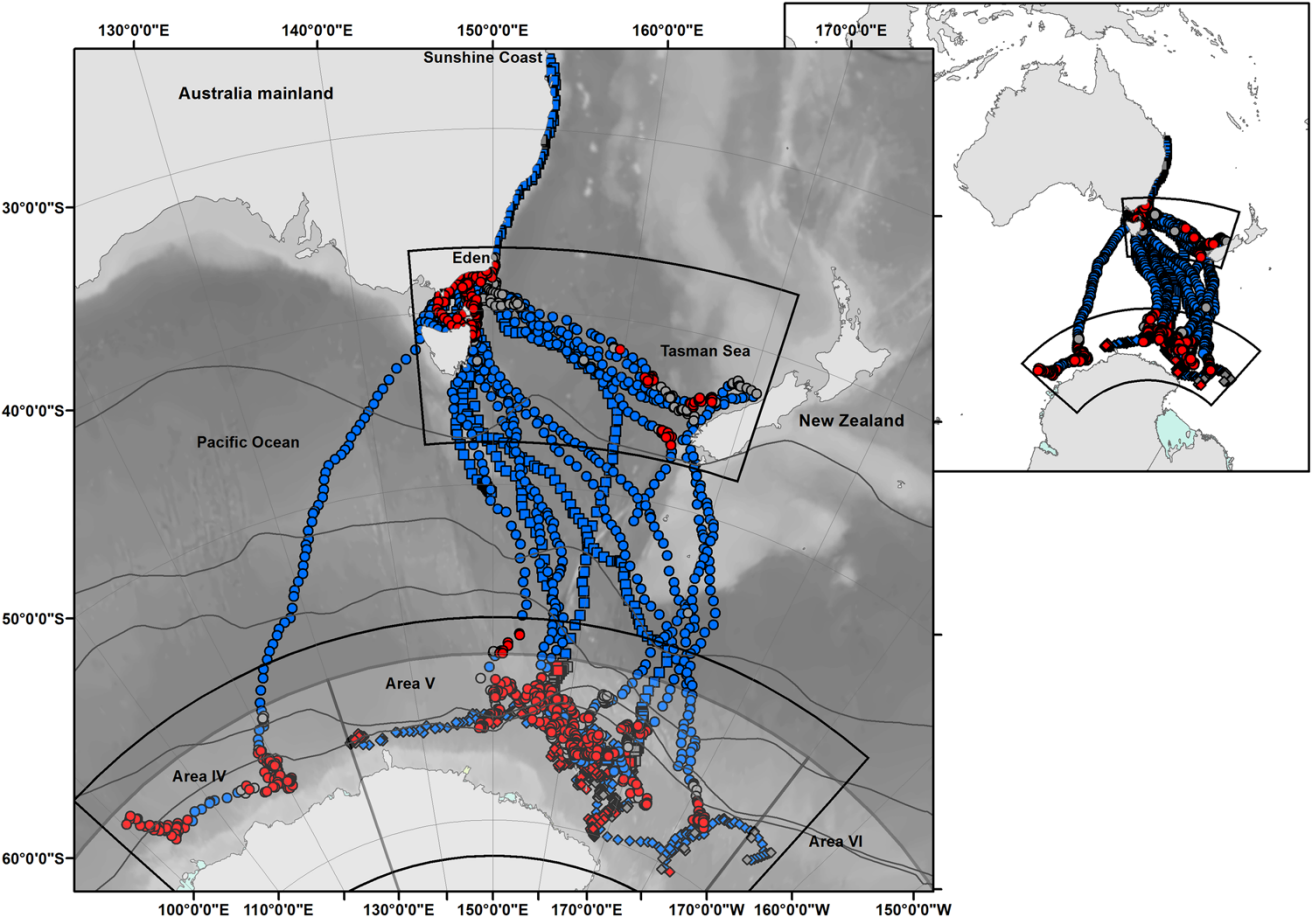
Migration pathways for 30 humpback whales satellite-tagged off the eastern coast of Australia. (Eden deployment – circles, Sunshine Coast deployment – squares) and in Antarctica (diamonds). (**a**) Shows tracks across the entire geographic range and depicts IWC Antarctic Management Areas IV, V and VI as white boxes. Location estimates from the state-space model are coloured according to the behavioural state estimate: ‘search’ (red), ‘transit’ (blue) and ‘uncertain’ (grey). Grey lines show climatological oceanic frontal positions^102^. Background shading indicates bathymetry derived from the GEBCO Digital Atlas^103,104^.

Thirty humpback whales were tracked during three austral summers (2008/09, 2009/10 and 2010/11; Fig. 1 and Table 1) over a period of 3 to 155 days with a mean (±SD) track duration of 50 ± 35 days (Table 2). Based on the filtered state-space location estimates, migrating whales travelled 2850 ± 1377 km (range: 103–5272 km, n = 21) from their tagging location, travelling a mean distance of 78 ± 22 km per day before crossing the 60 °S parallel into the Southern Ocean. On the Antarctic feeding grounds south of 60 °S, tracked whales covered a mean distance of 1885 ± 1567 km (range: 248–6315 km, n = 20), travelling 52 ± 18 km per day. In temperate waters and while migrating south, whales travelled at a speed of 3.32 ± 0.85 kmh^−1^ and when south of 60 °S slowed to 2.19 ± 0.74 kmh^−1^.

**Table 1:** Summary of satellite tags deployments and the resulting tracking information.

PTT	Gender	Tagging location	Deployment date (UTC)	First location (UTC)	Last location (UTC)	Track duration (days)	Transmitting days	Track distance (km)	Number of search patches	Total search time (days)	Max. search patch duration (days)	Min. search patch duration (days)	Search locations
88718	Male	AU-Eden	24/10/2008	24/10/2008	24/01/2009	93	92	5776	3	50	19	14	AU-EC (36), ANT (14)
88723	Unknown	AU-Eden	24/10/2008	24/10/2008	13/01/2009	82	81	5848	2	26	15	11	TS (11), ANT (15)
88732	Male	AU-Eden	24/10/2008	24/10/2008	27/11/2008	35	35	2571	1	8	8	8	TS (8)
88733	Female*	AU-Eden	24/10/2008	24/10/2008	4/12/2008	42	42	4042	0	NA	NA	NA	NA
88735	Male	AU-Eden	24/10/2008	24/10/2008	1/12/2008	39	38	1529	3	30	14	5	AU-EC (30)
88743	Male	AU-Eden	24/10/2008	24/10/2008	5/11/2008	13	13	583	0	NA	NA	NA	NA
88746	Male	AU-Eden	24/10/2008	24/10/2008	12/11/2008	20	20	1720	0	NA	NA	NA	NA
88725	Male	AU-Eden	25/10/2008	25/10/2008	13/01/2009	81	80	5520	3	61	29	15	AU-EC (29), ANT (32)
88744	Male	AU-Eden	25/10/2008	25/10/2008	27/10/2008	3	3	103	0	NA	NA	NA	NA
88745	Male	AU-Eden	25/10/2008	25/10/2008	12/11/2008	19	14	1429	0	NA	NA	NA	NA
88738	Male	AU-Eden	27/10/2008	27/10/2008	23/12/2008	58	58	4639	4	29	17	3	AU-EC (7), ANT (22)
88722	Female	AU-Eden	28/10/2008	28/10/2008	16/11/2008	20	20	1329	1	13	13	13	AU-EC (13)
88729	Female	AU-Eden	29/10/2008	29/10/2008	3/02/2009	98	97	7202	3	56	37	3	AU-EC (3), ANT (53)
88717	Female*	AU-Eden	31/10/2008	31/10/2008	29/11/2008	30	30	2025	1	8	8	8	TS (8)
88728	Female*	AU-Eden	31/10/2008	31/10/2008	1/02/2009	94	93	6312	3	37	16	5	ANT (37)
88741	Female	AU-Eden	1/11/2008	1/11/2008	4/04/2009	155	153	10324	6	93	28	4	AU-EC (4), ANT (89)
53348	Male	ANT	21/02/2010	21/02/2010	24/03/2010	32	26	1566	1	20	20	20	ANT (20)
53736	Female	ANT	21/02/2010	17/02/2010	6/04/2010	49	39	2486	1	9	9	9	ANT (9)
96385	Female	ANT	22/02/2010	22/02/2010	7/03/2010	14	14	487	1	5	5	5	ANT (5)
98138	Male	ANT	22/02/2010	21/02/2010	3/04/2010	42	42	1425	0	NA	NA	NA	NA
96403	Female	ANT	25/02/2010	26/02/2010	18/03/2010	21	18	1821	0	NA	NA	NA	NA
96386	Female	ANT	1/03/2010	1/03/2010	31/05/2010	92	92	4547	5	30	21	1	ANT (30)
96390	Female	ANT	8/03/2010	8/03/2010	28/03/2010	21	21	962	1	13	13	13	ANT (13)
96398	Male	ANT	8/03/2010	8/03/2010	22/04/2010	46	46	2643	5	25	7	3	ANT (25)
96412	Female	ANT	8/03/2010	8/03/2010	22/03/2010	15	15	860	2	9	5	4	ANT (9)
98139	Male	AU-SC	13/10/2010	13/10/2010	22/11/2010	41	41	2697	0	NA	NA	NA	NA
64235	Male	AU-SC	14/10/2010	14/10/2010	29/11/2010	47	47	4335	1	3	3	3	ANT (3)
98100	Female	AU-SC	14/10/2010	14/10/2010	1/12/2010	49	49	3580	0	NA	NA	NA	NA
98114	Male	AU-SC	14/10/2010	31/10/2010	9/12/2010	40	40	3225	0	NA	NA	NA	NA
98129	Female*	AU-SC	15/10/2010	15/10/2010	29/01/2011	107	104	6539	1	2	2	2	ANT (2)

For tagging location: AU-Eden is Eden, Australia; ANT is Antarctica; AU-SC is Sunshine Coast, Australia. For search location: AU-EC is east coast Australia, ANT is Antarctica, TS is Tasman Sea and number of days searching in each location is given in parentheses. *Indicates where gender was inferred by behaviour (with calf) at time of tagging (determined via biopsy otherwise).

**Table 2:** Summary movement statistics for thirty satellite tracked humpback whales.

	Number of whales tagged	Track length (days; mean ± SD)	Track distance (km; mean ± SD)	Initial migratory trajectory			Temperate search patches		Travelled south of 60 °S	Antarctic search patches
				South	West (crossing the 146 °E meridian)	East (crossing the 160 °E meridian)	East coast Australia: Eden, Bass Strait and Tasmania	Tasman Sea		
AU-Eden*	16	55 ± 42	3809 ± 2854	8	1	6	7	3	8	7
AU-SC	5	57 ± 28	4075 ± 1500	4	0	1	0	0	3	2
ANT	9	37 ± 25	1866 ± 1235	NA	NA	NA	NA	NA	9	7
Total	30	50 ± 35	3271 ± 2414	12 (57%)	1 (5%)	7 (33%)	7 (33%)	3 (14%)	20 (67%)	16 (53%)

Numbers indicate individual animals, with percentages (%) given in parentheses. Trajectory and temperate search patch statistics apply only to whales tagged off Eden and Sunshine Coast, Australia. *One tag failed before transmitting a clear migratory trajectory.

**Figure 2:**
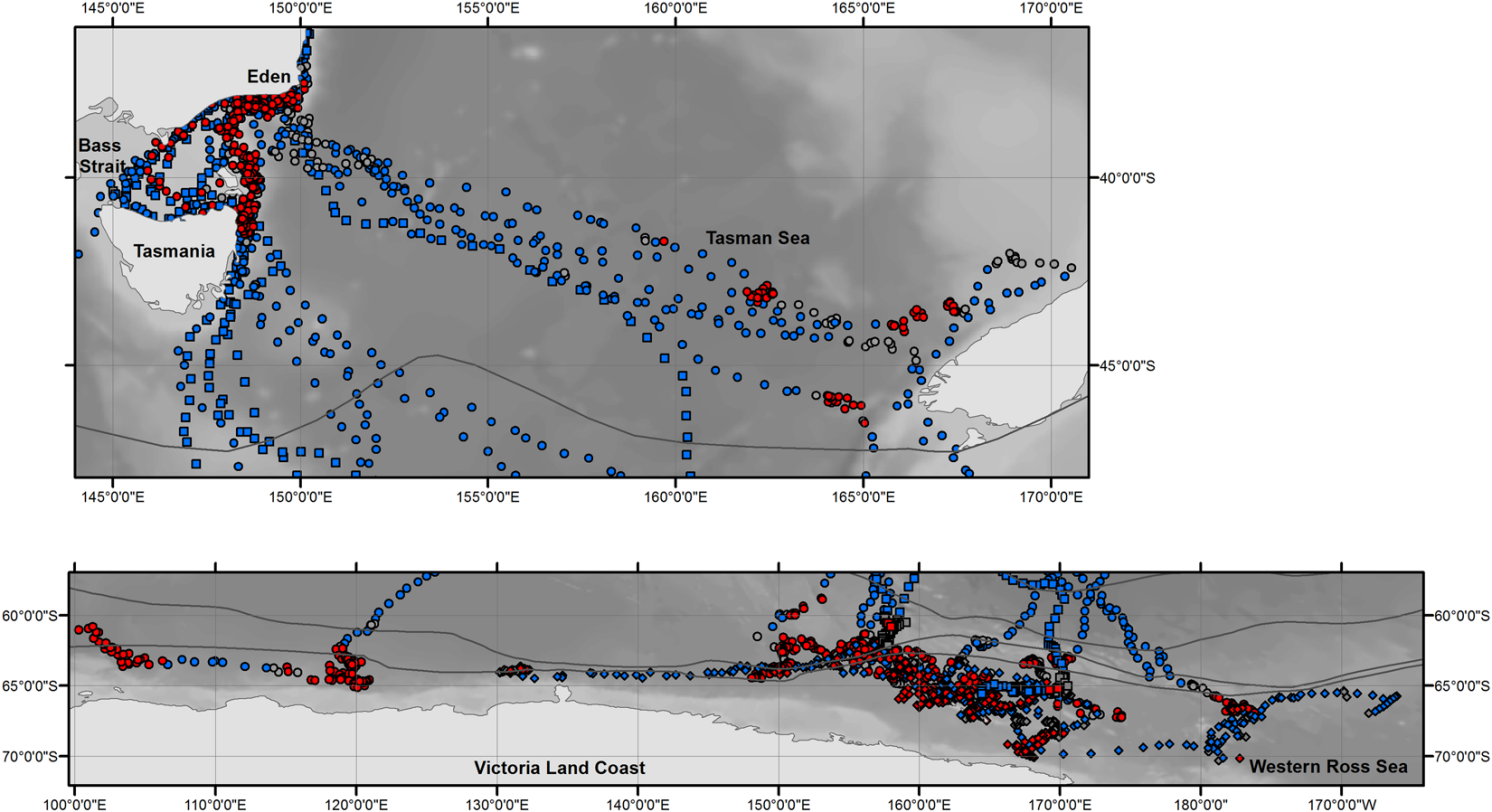
Detail of inset areas shown in Fig. 1. (**a**) The temperate, and (**b**) the Antarctic foraging zones outlined as black boxes in Fig. 1. Location estimates from the state-space model are coloured according to the behavioural state estimate: ‘search’ (red), ‘transit’ (blue) and ‘uncertain’ (grey). Grey lines show climatological oceanic frontal positions^102^. Background shading indicates bathymetry derived from the GEBCO Digital Atlas^103^.

The 21 whales tagged off the eastern Australian coast migrated south along the coastline and across the Bass Strait (separating mainland Australia and Tasmania) during the month of October (Fig. 2a). Throughout November, 12 whales migrated south via the east coast of Tasmania (one tag failed prior). One whale migrated via the west coast of Tasmania and continued in a south westerly direction into the Pacific Ocean then moved onto the Antarctic feeding grounds (Figs 1, 2b and Table 2). Seven whales travelled eastwards into the Tasman Sea crossing the 160 °E meridian whilst still in temperate waters (Fig. 2a). Three of these whales spent time off the south west coast of New Zealand’s South Island (14^th^ to the 29^th^ November 2008) while the other individuals continued transit into the Southern Ocean.

In total eleven humpback whales tagged in east Australian waters travelled south of 60 °S (Fig. 2b), with first arrival dates of 29^th^ November 2008 (n = 8) and the 21^st^ November 2010 (n = 3), respectively. The mean date of arrival at 60 °S was the 10^th^ December in 2008 (median: 7^th^ December 2008) and the 4^th^ December in 2010 (median: 23^rd^ November 2010). All humpback whales with transmitting tags were in the Antarctic feeding grounds by January (the latest arrival was an Eden tagged whale 88718 on the 1^st^ January 2009) where location data continued to be transmitted until May (Antarctic tagged whale 96386 – last data transmitted on 31^st^ May 2010).

### Whale behaviour

The state-space model clearly distinguished between two behavioural states for the migrating whales (nominally, search and transit; Table 3). The main parameters governing the movement processes (θ – turn angles, and γ – movement persistence; see^18^) were very well discriminated for the two states. The turn angles clearly showed frequent reversals during search (mean turning angles concentrated around 176° to 192°) as opposed to few when transiting (mean turning angles concentrated near zero, around −1° to 1.5°; Table 3). In general, credible intervals around parameter estimates were tight, with no overlap in parameter estimates between the two states. The estimated movement persistence was notably higher for those animals tagged within east Australian waters (γ_1_ > 0.8), which undertook the longest migrations, as compared with those animals tagged on the Antarctic foraging grounds (γ_1_ ~ 0.5). Overall, whales spent on average 29.7 ± 27.8% (0–76.9%) of their time in search behaviour; 14.1 ± 22.7% when north of 60 °S (n = 21) and 29.8 ± 23.6% (n = 20) when south of 60 °S. There was relatively low uncertainty in the behavioural state estimates for the two Australian deployment campaigns with 15.6% (Eden) and 13.7% (Sunshine Coast) of estimates falling between 1.25 and 1.75; this was somewhat higher for the Antarctic deployment at 34.6%.

**Table 3:** Posterior sample means and 95% credible intervals (CIs) for movement parameters estimated using a hierarchical state-space behavioural switching model.

Deployment campaign	N	State 1 ‘transit’				State 2 ‘search’			
		γ_1_		θ_1_		γ_2_		θ_2_	
		Mean	95% CI	Mean	95% CI	Mean	95% CI	Mean	95% CI
ANT	9	0.515	0.428–0.625	0.352	0.214–0.470	−0.018	−0.098–0.065	3.294	3.111–3.494
AU-Eden	16	0.821	0.785–0.856	0.111	0.045–0.177	0.021	−0.012–0.053	3.074	2.692–3.425
AU-SC	5	0.837	0.797–0.876	0.160	0.003–0.664	0.027	−0.017–0.071	3.350	1.779–4.813

Turning angles (θ) are given in radians, the movement persistence parameter (γ) is the correlation in speed and direction. Subscripts indicate behavioural state. The state-space model was run on each campaign separately, with N indicating each sample size (number of whales).

Ten out of 21 animals (47.6%) tagged during migration in east Australian waters had ‘search’ behaviour identified at locations within temperate waters; these were all animals tagged off Eden. Three of these whales undertook short search periods (of three days) near the tagging location from October 24, 2008, whilst four of these whales undertook search extending into the Bass Strait and/or along the east coast of Tasmania ranging in patch duration from four to 35 days (Fig. 2a, Tables 1 and 2). For those whales that travelled eastwards, search patches of between seven and 10 days were located in the Tasman Sea and off the south west coast of New Zealand’s South Island (n = 3; Fig. 2a, Tables 1 and 2).

Within the Antarctic feeding grounds (Fig. 2b) search behaviour was documented for 20 whales over three consecutive austral summers (Tables 1 and 3). Search behaviour was detected soon after animals tagged off eastern Australia (2008/09 and 2010/11) arrived south of 60 °S, with a mean start date for inferred foraging on the 17^th^ December in 2008 (median: 11^th^ December 2008; n = 7) and the 18^th^ of December in 2010 (median: 18^th^ December 2010; n = 2). The majority of search behaviour was concentrated between 145–175 °E (Fig. 2b). Over all seasons, from whales tagged both on migration and on their feeding grounds, there are three small areas containing both transit and search behaviours that overlap or are closely adjacent between years. Two of these areas are located at approximately 60.5 °S to 61.5 °S and 158 °E (Eden and Sunshine Coast deployments – austral summer 2008/09 and 2010/11 respectively), and at 64.7 °S to 66.4 °S and 169.5 °E (Eden, Antarctic and Sunshine Coast deployments – austral summers 2008/09, 2009/10 and 2010/11 respectively). The third area was farther east, at 65.5 °S to 67.2 °S near 178 °W (Eden and Antarctic deployments – austral summers 2008/09 and 2009/10 respectively). This coherence in behaviour between years suggests the potential for persistent space use and well established migratory pathways.

### Behaviour-environment associations

**Figure 3:**
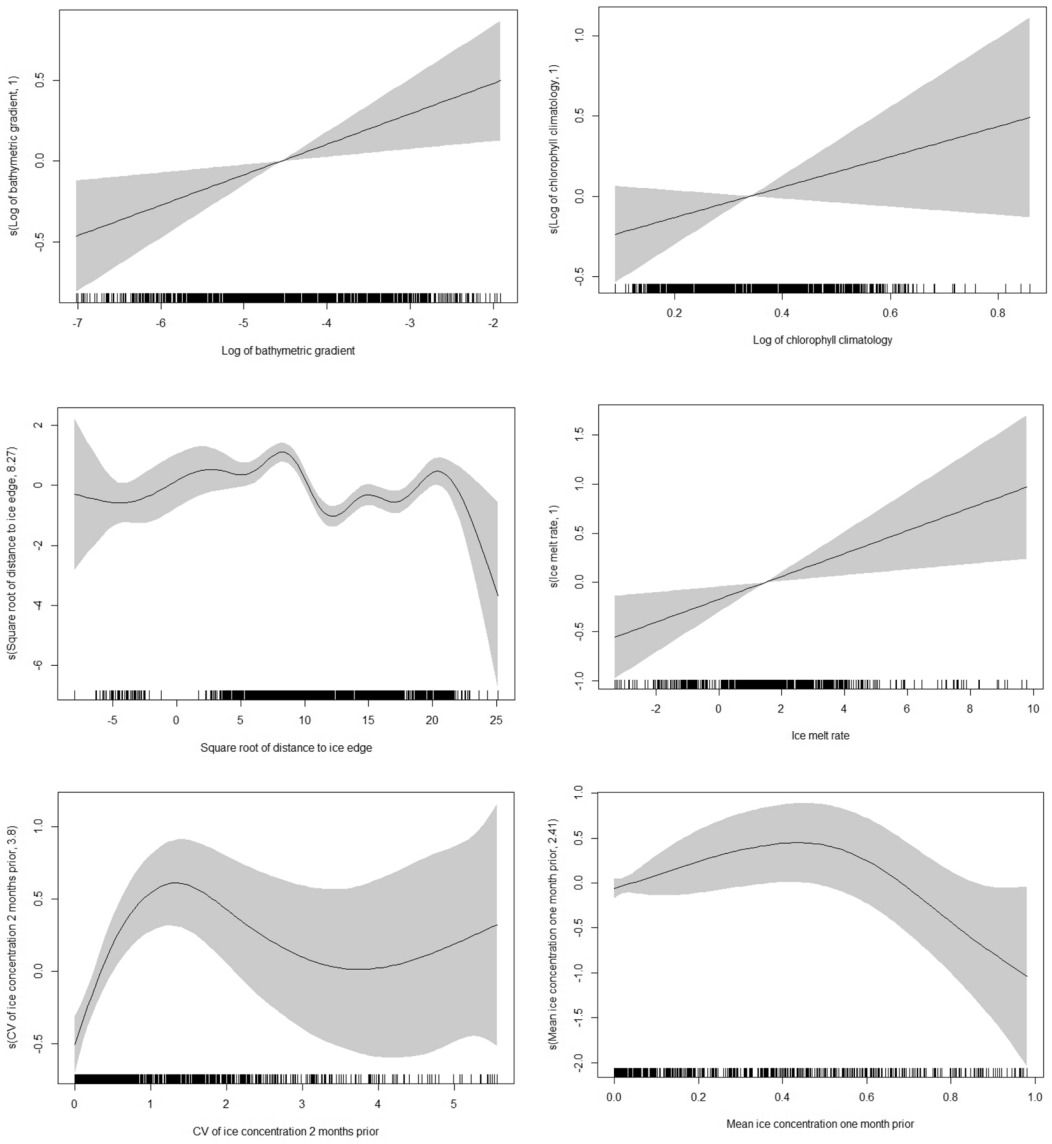
Smooths of generalized additive mixed modelling. (GAMM) terms showing the influence of environmental variables on whale ‘search’ behaviour (*b*). Locations of observations are shown as tick marks on the *x*-axes. Solid lines are the estimates of the smooths, grey areas indicate standard errors of the estimated smooths. The y-axis indicates the effect of the smooth function of each covariate upon the probability of being in ‘search’ behaviour; with a lower (higher) value indicating reduced (increased) probability. (**a**) Bathymetric gradient, (**b**) summer chlorophyll climatology, (**c**) distance to ice edge, (**d**) ice melt rate, (**e**) coefficient of variation (CV) of ice concentration two months prior, and (**f**) mean ice concentration one month prior.

Whale behavioural state (search and transit) varied in relation to a suite of environmental variables (*p*-values < 0.05, Table 4; full model summary in Supplementary S2, Table S2.3). The GAMM smooths indicate that humpback whales were more likely to adopt search behaviour at higher bathymetric gradients (Fig. 3a) and where ice melt rate was high (Fig. 3d). Non-linear relationships indicated a higher occurrence of search behaviour approximately 65 km from the ice edge (Fig. 3c), where ice concentration variability was moderately high 2 months prior (Fig. 3e) and at mid-range ice concentrations one month prior (Fig. 3f). Search behaviour was not significantly related to the seasonal chlorophyll-a climatologies (Fig. 3b), nor was deployment campaign a significant predictor.

**Table 4:** Significance of environmental predictors influencing whale behaviour.

Environmental variable	*p*-value	Number of times significant/100
*log bathyg*	0.007	20
*log CHLa*	0.114	2
*sqrt dist ice*	<0.001	91
*melt rate*	0.008	73
*ice cv lag 2*	<0.001	96
*ice mn lag 1*	0.026	26

*P*-values indicate approximate significance of GAMM smooth terms based on Chi-sq. statistics. Second column indicates the number of times each environmental variable^105–109^ was determined to be significant (based on *p*-value < 0.05) under the resampling procedure (n = 100 iterations, resampling randomly from the state-space model posterior estimates for behavioural state and location).

The resampling procedure examined the retention of model predictors given the uncertainty inherent in the behavioural state and location estimates. This showed that inclusion of the three ice-related variables was the most highly resilient to uncertainty (Table 4). The ice CV and the distance to ice edge were retained as significant in over 95% and 90% of the models fit during the resampling procedure, respectively. Ice melt rate was retained as significant in over 70% of the resampling models whereas the other variables were less resilient (more sensitive) to uncertainty. This result highlights the prominent role that features of the marginal ice zone play in influencing the search behaviour of humpback whales.

**Figure 4:**
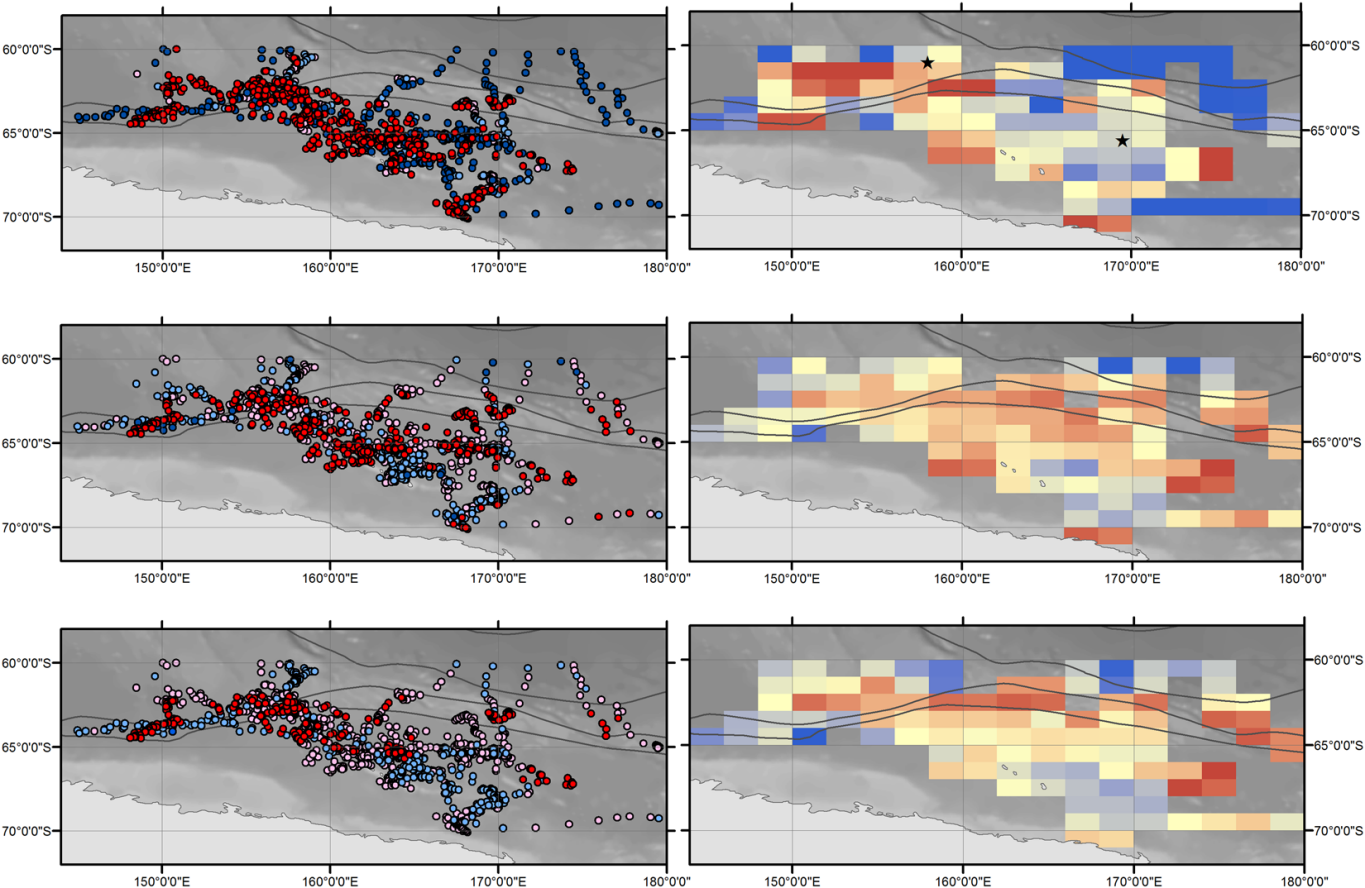
Maps showing whale behavioural state estimates. (**a**) Behavioural state estimates from the hierarchical state-space switching model. Panel focusses on the area of most concentrated search behaviour between 145–175 °E. Predicted probabilities of being in search behaviour obtained from (**b**) the GAMM using environmental predictors, and (**c**) averaging the GAMM predictions (n = 100) across the resampling procedure (see Methods). Colours represent the gradient from transit (P < 0.25, dark blue) through to search (P > 0.75, red) at 0.25 intervals. Right hand panels: (**d**,**e** and **f**) spatially aggregate these probabilities into 1° latitude ×2° longitude grid cells by averaging across individual whales. Higher mean probabilities of search behaviour shown as warmer colours. The star symbols in (**d**) represent areas of persistent use between tracking years. Background shading indicates bathymetry derived from the GEBCO Digital Atlas^103^.

The concentration of search behaviour occurring between 145–175 °E (Fig. 4a) was generally well captured using our environmental model to predict whale movement behaviour (Fig. 4b). The predicted probabilities of search behaviour were typically reduced when uncertainty was incorporated via the resampling procedure, although the spatial patterns remain similar overall (Fig. 4c). When spatially aggregated these predictions may give an indication of habitat areas important for foraging (i.e. high mean probabilities of whales undertaking search behaviour, shown as warmer colours in Fig. 4d–f), opening the possibility for more broad scale habitat predictions on the basis of environmental drivers. For ease of interpretation, the two areas of persistent space use that are located between 145–175 °E and reported within ‘Whale behaviour’ are represented by the star symbol in Fig. 4d.

## Discussion

The capability of satellite tags to detail whale movements is markedly building our understanding of how whales move and interact with their environment throughout important migration pathways^22,28^. This novel information ultimately plays an important role in conservation and management^11,29^. The tag-derived movements reported here substantially increase our current knowledge of the east Australian humpback whale population by revealing new movement patterns, unidentified temperate feeding locations and providing the first description of the environmental predictors that characterise key foraging habitat in Antarctica.

Satellite tracked humpback whales tagged off eastern Australia travelled south along three different migratory trajectories, detailed for the first time in this study. The majority of animals initially travelled southwards, following the eastern mainland and Tasmanian coastlines. One third of these whales headed in an easterly direction into the Tasman Sea. Of these seven individuals, three tags transmitted long enough to record movement as far as 165 °E, and the New Zealand coastline, before changing direction to transit to Antarctica. The simple information gained from historic Discovery marks hints at these migratory routes, with humpback whales marked off eastern Australia then captured off New Zealand and Antarctica (IWC Antarctic management areas IV and V^30^). A movement not previously represented within the Discovery mark datasets is that of a lone individual who migrated along a westward trajectory through the Bass Strait and onto Antarctica, venturing as far west as 166 °W (management area VI).

The state-space model indicated areas of search behaviour within temperate habitat for almost half (47.6%) of the individuals tagged off eastern Australia. Temperate search behaviour was demonstrated only amongst those individuals tagged off Eden, Australia where supplemental feeding on small schooling fish and krill has been regularly reported^31,32^. Whilst apparent search might relate to foraging, resting, breeding or other behaviours^33–35^, seven humpback whales were directly observed to be feeding off Eden at the time of tagging. Additional areas not previously associated with supplemental feeding were identified through the Bass Strait, along the east coast of Tasmania, and within the eastern Tasman Sea off the southern New Zealand coastline. This temperate search behaviour persisted for longer than 30 days for three individuals within the Bass Strait/Tasmanian region and for 7–10 days in the Tasman Sea off New Zealand.

Traditionally, humpback whales are considered to adopt the “feast or famine” approach to migration that is typical of baleen whales: fasting when not occupying high latitude feeding grounds^30^. However by temporarily suspending migration to forage, these whales may be able to meet up to 3.4 times their daily energy requirements. This may supplement the energy needed for migration and, in the process, begin to refuel energy reserves prior to reaching their Antarctic feeding grounds^31^. Riekkola *et al*.^29^ inferred supplemental feeding in endangered Oceania humpback whales and hypothesised that supplemental feeding may occur when energetic requirements are not being met on Antarctic feeding grounds. Supplemental feeding has also recently been noted for individuals from other Southern hemisphere humpback whale populations including those off the west coast of Africa^36,37^ and both the east^38^ and west^39^ coasts of South America. Stable isotope analyses of baleen whale plates indicate that supplemental feeding may actually be quite a common strategy for east Australian humpback whales^40^.

The primary Antarctic foraging grounds of east Australian humpback whales was historically considered to be between 130 °E and 170 °W (IWC Antarctic management area V) as determined through Discovery mark recoveries^30^. More recently, photo identification data has added further weight to the importance of IWC Antarctic management area V^41,42^. Our study largely supports this, with the majority of search behaviour of satellite tagged animals occurring between 145°–175 °E. However the tracking data demonstrates that east Australian humpback whales do undertake search behaviour across a wider range between 100 °E and 165 °W; i.e., encompassing IWC Antarctic management areas IV, V and VI. Search patches within Antarctic waters persisted for up to 37 days with individuals transiting amongst up to five patches. This type of movement path, with periods of more tortuous track segments connected by relatively directed track segments, is consistent with those exhibited by other satellite tagged humpback whales foraging on Antarctic feeding grounds off the West Antarctic Peninsula^43–45^, as well as within the Ross, Amundsen and Bellingshausen Seas^29^. Marine prey resources are patchily distributed at multiple spatial scales^46^. So, following the major migration transit south to the polar feeding grounds, animals will typically still need to search between dynamic favourable forage patches within and around the sea ice and Antarctic shelf regions (see e.g.^47^).

Our study identified important environmental predictors characterising Antarctic foraging habitat of east Australian humpback whales - high variability (in relation to the mean) in ice concentration two months prior to arrival, at a distance of approximately 65 km from the ice edge, and high ice melt rates at the time of ice retreat. These factors are clearly associated with the marginal ice zone. Essentially these humpback whales foraged where the sea ice was present two months prior. This is the first time this area, located off the Victoria Land coast and in the western Ross Sea, has been clearly identified as core foraging habitat for the east Australian humpback whale population, and defined in terms of key biophysical characteristics. The chlorophyll climatology we constructed as a proxy for primary oceanic productivity was only found to be a significant environment predictor in 2% of the statistical models. This is likely due in part to the inability of satellites to measure productivity of closely ice-associated habitats^48^. Additionally, persistent cloud cover necessitates averaging of remotely sensed chlorophyll measurements (here across a three month period); leading to the loss of temporal information.

The marginal ice zone is defined as the area of transition from dense pack ice (up to 100% cover) through to no ice cover^49^ and is outer pack ice that persists for ≤100 days in a climatological sense^50^. It is perhaps not surprising that the marginal ice zone and more specifically, low mean and high variance in ice concentration two months prior, plays an important role in conditioning foraging habitat. Various modelled estimates indicate that it takes approximately 15 to 20 days^51^, around 30 days^52^ and less than 90 days^53^ for the biological cascade that results from the release of new production (by way of ice melt) to trigger the accumulation of zooplankton grazers such as krill on which baleen whales foraging in Antarctica depend^54^. In fact, productivity peaks within the marginal ice zone one to two months following the point at which maximum open water in the area is achieved, which is a direct reflection of the time it takes for phytoplankton blooms to fully respond to newly created ice-free waters^55^. Rapid ice retreat has also been shown to enhance production, which concentrates secondary producers and their vertebrate predators^54,56^. However, changing ice dynamics are not favourable for all Antarctic marine predators (for e.g.^57^).

Large spatio-temporal variability in sea ice seasonality is a fundamental feature of East Antarctica^50^ and the area in which the East Australian humpback whales forage. The high variability in the sea ice season between 100 °E and 145 °E occurs primarily in the marginal ice zone region (see Fig. 5c)^50^. East of 145 °E, high variability in the ice season occurs across the entire ice covered region with the exception of the coastal fast ice zone. Despite such large variability, the foraging habitat of the east Australian humpback whale population has undergone a trend of increasing ice season duration over a 30 year period, through earlier advance and later retreat of the ice edge (see Fig. 6)^50^. The region east of 145 °E undergoes earlier and rapid ice edge advance with strong ice production occurring in autumn. Due to fewer open water days there has been an overall trend of decreasing sea surface temperature and decreasing net primary productivity, but also a small area of net primary productivity increase located at approximately 64 °S to 66 °S and 160 °E to 165 °E^58^.

The Antarctic foraging habitat of east Australian humpback whales could also be habitat favourable for krill recruitment and success. Traditionally, increased ice season duration and associated winter ice has been thought to favour krill maturation by providing a winter, ice algae food supply resulting in a high recruitment rate from the spawning season^59,60^. However, recent evidence suggests that at least in East Antarctica, this relationship may not be so direct and that the winter pack ice may actually be a food-poor habitat for krill^61^. The marginal ice zone provides a better feeding habitat for krill due to the presence of light, nutrients, grinding ice floes and proximity to open ocean/waves which promote high larval krill growth rates. The complex habitat structure of the marginal ice zone further provides protection important for larval krill survival. Phytoplankton availability in autumn may also govern krill recruitment success with early ice formation in autumn separating adult and larval krill and reducing food competition. As such, time lags in ice-related environmental predictors that date back to the previous autumn may need to be considered in future modelling attempts.

The IWC’s International Decade for Cetacean Research (IDCR) and Southern Ocean Whale Ecosystem Research (SOWER) sightings surveys^62,63^ and whaling catch records^63,64^ demonstrate that historically, humpback whales were located (sighted and captured) in relatively high numbers within the same area identified as foraging habitat for the humpback whales tagged during 2008–2010. Our findings showed notably persistent space use, such that different whales moved through the same location or occupied habitat immediately adjacent over three consecutive austral summers. Site fidelity, or persistent space use, has been observed for other humpback whale populations^39,45,65,66^. In the patchy marine environment, foraging site fidelity may be attributed to the interplay of habitat quality and predictability; ultimately, familiarity with foraging habitat may present significant ecological benefits over the long term^67,68^. Additionally, for humpback whales, foraging site fidelity is maternally directed with individuals returning to foraging sites that, historically, they first visited with their mother prior to weaning^69–71^. This behavioural mechanism can act on population structure at an evolutionary time scale^72^ and may contribute to the lack of recovery for some whale populations that were nearly extirpated due to whaling^73^.

East Australian humpback whales were hunted along both their migratory corridors and upon their Antarctic feeding grounds during the 20^th^ century^25^, and may have numbered just 104 individuals when commercial whaling ceased in 1963^26^. This population, assigned the nomenclature E1, is considered to be one of three meta-populations that comprise population E^74^. The other two meta-populations (New Caledonia E2, and Tonga E3) together with individuals from the central South Pacific collectively comprise the Oceania population. The rapid recovery rate of the east Australian population is just shy of the theoretical upper bound of population increase^75^ at 11.1% (95% CI of 10.6–11.3%^27^). Whilst the E1 post-exploitation recovery is strong at between 58–98% recovered^27^, the Oceania group remains listed as endangered by the International Union for Conservation of Nature^76^ and is estimated to be well below 50% of pre-exploitation population size^29^. This group, which was decimated by illegal hunting of Soviet whalers^77^, mainly forage farther east throughout IWC Antarctic Management Areas V, VI and I (approximately 180 °E to 90 °W)^29,78^. Tracking data demonstrate a small degree of overlap in foraging range at the eastward limit of the satellite tracks presented in this study. Clearly these populations can mix on their foraging grounds^78^ which highlights the possibility that the rapid recovery of east Australian humpback whales may only be partly due to forage conditions (access to quality foraging grounds and supplemental feeding). Conversely, the poor recovery of the Oceania population may simply just be the result of their near extirpation. However, Clapham and Zerbini^79^ hypothesise that the high population growth rate of east Australian humpback whales could be due to temporary immigration by Oceania humpbacks as a consequence of a mating system that results in whales migrating from low density to high density breeding grounds. In light of the annual pregnancy rates recently reported for two Southern Hemisphere humpback whale populations^29,80^, a revisit of appropriate calving intervals along with further genetic analyses are required to determine the validity of this hypothesis.

The east Australian humpback whale population is perhaps the best monitored population of Antarctic krill consumers in the world, with post-whaling surveys initiated in 1978 and repeated every one to three years since^27^. Easily counted and sampled whilst on their tropical breeding ground, this population could act as a “sentinel species” providing cost-effective monitoring of the Antarctic sea ice ecosystem^27,81^. Significant changes are already occurring in key Antarctic forage habitat, with evidence of long-term changes in the sea ice environment^50^ and associated declines in ocean temperature and net primary productivity^58^. The capacity for these long lived, large animals with late reproductive age to respond to change is not known, but presumably currently being challenged by rapid environmental change. The future brings potential impacts of even larger scale changes: for example, the possibility of a complete collapse in Antarctic krill populations due to ocean acidification by 2300^82^. Highly mobile species which rely on different habitats during different life history stages are subject to multiple and varied threats across their range, which makes it difficult to understand and predict their ability to adapt^83,84^. Certainly as this population approaches recovery, ongoing monitoring will be required to identify and address the various impacts of human and economic expansion on migratory pathways and breeding habitat^85^. However, if we consider that migration has evolved as a successful strategy to manage environmental variability, and persisted as a behaviour throughout global change over millions of years^84^, then for east Australian humpback whales the act of migration may facilitate their ability to respond to change.

## Methods

### Tag deployments and tracking information

Satellite tags were deployed on adult humpback whales with a modified version of the Air Rocket Transmitter System ARTS, Restech^86^ and a purpose-designed projectile carrier at a pressure of 7.5–10 bar. A custom-designed, 80 mm anchor section is attached to a stainless steel cylindrical housing containing a location-only transmitter (SPOT-5 by Wildlife Computers, Redmond, Washington, USA and Kiwisat 202 Cricket by Sirtrack, Havelock North, New Zealand). This superseded anchor design resulted in the anchor section disarticulating upon deployment in order to achieve improved tag retention times while minimising impact^87^. The tags were sterilised with ethylene oxide prior to deployment and implanted up to 290 mm into the skin, blubber, interfacial layers and outer muscle mass of the whale.

Upon deployment, a small amount of skin and blubber was simultaneously collected for genetic analyses. These were collected using a biopsy dart fired from a modified 0.22 Paxarms system^88^. Biopsy samples were stored in 70% ethanol and DNA subsequently extracted using a Tissue DNA purification kit for the Maxwell 16 DNA extraction robot (Promega Corporation). The sexes of the tagged whales were determined using a 5′ exonuclease assay of the polymorphisms in the sex-linked Zinc Finger genes as described by Morin *et al*.^89^.

Tags were deployed during three austral summers: 2008/09, 2009/10 and 2010/11. The two Australian deployment campaigns were on individuals undertaking their southern migration along the east coast near Eden (37.15 °S, 150.07 °E; n = 16) between October and November 2008 and off the Sunshine Coast (26.51 °S, 153.17 °E; n = 5) during October 2010. Satellite tags were also deployed on individuals whilst on their Antarctic feeding grounds during February and March 2010 (west of the Balleny Islands, approximately 67.25 °S, 152.71 °E; n = 9).

Tags were programmed to transmit to the Argos satellite system at various duty cycles and repetition rates for a maximum of 720 transmissions per day (Table S1). These transmissions are relayed to processing centres which calculate the transmitter’s location by measuring the Doppler Effect on transmission frequency. Transmitted data were processed using a least squares analysis and each location was assigned an estimated error and one of seven associated location classes (see^90^).

### Hierarchical switching state-space model

Argos location data (described in^91–93^) from whales in each deployment campaign (Eden: n = 16; Antarctica: n = 9; Sunshine Coast: n = 5) was entered into a hierarchical switching state-space model which enables joint estimation over multiple individuals (hSSSM^94^). This model both accounts for spatial uncertainty in Argos location estimates (via an observation model) and estimates two discrete (categorical) behavioural states (via a movement process model). The hierarchical structure assumes that all individuals move according to a correlated random walk but that that the movement of each individual is characterised by a different diffusivity^17^. Using a Bayesian approach, the movement process parameters governing each state are estimated: the mean turning angles (θ) and a movement persistence parameter (i.e., the autocorrelation in speed and direction – γ). Here, we nominally label the two states as transit (indicating more directed travel) and search (indicating more localised residency) with the latter putatively indicating foraging behaviour although alternate behaviours, e.g., resting, breeding or social activities may be relevant for particular species in certain areas^33,34,95^.

For each of the deployment campaigns separately, the hSSSM was fit across animals (AU-Eden: n = 16; AU-SC: n = 5; ANT: n = 9) using a 12 h time step. The hSSSM is fit via Markov chain Monte Carlo (MCMC) simulation implemented in JAGS using the R^96^ package *bsam*^17,18,94^. Two MCMC chains were run in parallel for a total of 100,000 samples each with the first 50,000 discarded as burn-in and the remaining samples thinned to every 50^th^ sample to address autocorrelation. This yielded a total of 1000 samples retained from the joint posterior. Each MCMC sample provides a discrete behavioural state estimate (*b*, where 1 = ‘transit’, 2 = ‘search’) associated with each location state (longitude, latitude) estimate. From the posterior, the most probable (discrete) behavioural state can then be evaluated for each location, as well as a summary made across all samples e.g., as a posterior mean (giving a value continuous between 1 and 2). For mapping purposes the behavioural state shown is the mean posterior estimate and following Jonsen *et al*.^95^ we assign estimates <1.25 as ‘transit’ (blue) and >1.75 as ‘search’ (red). All estimates between 1.25 and 1.75 are shown as ‘uncertain’ (grey). To calculate search patch duration we follow Bailey *et al*.^33^ where a patch is comprised of successive location estimates with an estimate greater than 1.75, ending when 3 or more consecutive location estimates have an estimate below 1.75.

### Statistical model for behaviour-environment associations

To investigate potential environmental influences on whale search behaviour whilst on the Antarctic forage grounds we used generalized additive mixed models (GAMMs^97^). The most probable discrete behavioural state estimate (*b*, where 1 = ‘transit’, 2 = ‘search’) was modelled in response to environmental predictors selected based on their potential to influence the search behaviour of humpbacks whales by concentrating prey. The environmental variables examined, with full details of the data sources and resolution are given in Supplementary S2, Table S2.1. These predictors may act as physical habitat boundaries, influence biological productivity and/or characterise recent ice history. Preliminary investigations examined candidate ice variables at both a one month and two month lag (exploratory models presented in Supplementary S2, Table S2.2). Collinearity amongst predictors^98^ was assessed using variance inflation factors (ensuring VIF <3) and correlation coefficients (ensuring *r* < 0.8 – Pearson correlation coefficient). The environmental variables included in the final full GAMM (see Supplementary S2, Table S2.3 for full results) were: bathymetric gradient (*bathyg*), chlorophyll climatology (*CHLa*), distance to ice edge (*dist ice*), ice melt rate (*melt rate*), the coefficient of variation in ice concentration two months prior (*ice cv lag 2*) and mean ice concentration one month prior (*ice mn lag 1*). Deployment campaign was also included as a categorical predictor. We examined spatial and temporal autocorrelation in the Supplementary material (see Supplementary S2, S2.4) and included an animation (see Supplementary S3) depicting each monthly coefficient of variation in ice concentration along with the state space modelled location estimations two months prior, one month prior and throughout the same month to aid in interpretation of this *ice cv lag 2* variable.

There is uncertainty inherent in the behavioural state estimate (here the response variable in our GAMMs) for each whale location, and also for each location estimate itself. It is particularly relevant to propagate location uncertainty when sampling environmental variables from spatially gridded remote-sensing data, although this is relatively rarely considered (but see^99^). To account for the uncertainty in both behavioural states and locations we resampled from the state-space model posterior estimates, thereby adopting a type of sensitivity analysis or bootstrap approach to fitting the GAMMs; this approach to accounting for measurement error is also called multiple imputation. We fit the first GAMM using the hSSSM behavioural state and location estimates summarised as the posterior mean for each location (n = 1442). We then randomly sampled the retained MCMC chains to obtain 100 possible realisations (50 per chain) of these 1442 behavioural and location state estimates.

These new samples drawn from the posteriors were then used to refit the above model 100 times. The discrete behavioural state (*b*) estimate at each location of the new track realisation was modelled against the relevant set of environmental predictor variables (also extracted at the new posterior sample locations). This enabled assessment of the strength of environmental influences accounting for uncertainty in the state-space model posterior estimates for behavioural state and location. We report the number of times each environmental variable was determined to be significant (based on *p*-value < 0.05) under the resampling procedure (n = 100 iterations). *P*-values indicate approximate significance of GAMM smooth terms based on Chi-sq. statistics. The GAMM was fit using a binomial family with a logit link, with individual whale included as a random effect. The GAMM allows for the relationships between behaviour and environmental predictors to be flexible and non-linear. All statistical models were fit using the *gamm4*^97^ library in R^96^ which makes use of the modular fitting functions provided by *lme4*^100^ and is appropriate for binary data. *gamm4*^97^ follows the approach taken by package *mgcv*^101^ and represents the smooths using R penalized regression spline type smoothers, of moderate rank. We focussed on the Antarctic forage grounds, restricting our behavioural analysis to only those locations at or south of 60 °S (n = 20 whales, N = 1442 locations).

This study was carried out in strict accordance with the approvals and conditions of the Antarctic Animal Ethics Committee for Australian Antarctic Science project 2941. Fieldwork was undertaken with the permission of the Australian Government under EPBC permits 2007–006 and 2007–007.

## Electronic supplementary material

Supplementary 1,2 and 3

Supplementary 3

## Data Availability

See^91–93^ for permanent links to the satellite tracking data analysed in this study which is held by the Australian Antarctic Data Centre at https://data.aad.gov.au/.
