# Supplementary material for: Humpback whale migrations to Antarctic summer foraging grounds through the southwest Pacific Ocean
Source: Sci Rep. 2018 Aug 17;8:12333. doi: 10.1038/s41598-018-30748-4 (PMC6098068; doi:10.1038/s41598-018-30748-4)
Supplement: Supplementary file 1 — Supplementary 1,2 and 3 [file 41598_2018_30748_MOESM1_ESM.pdf]

Humpback whale migrations to Antarctic summer foraging grounds through the southwest Pacific Ocean

V Andrews-Goff1, S Bestley, NJ Gales, S Laverick, D Paton, AM Polanowski, N Schmitt and MC Double

Supplementary S1. Details of satellite tag deployments.

Satellite tags deployed on east Australian humpback whales – PTT is the unique Argos identifier, manufacturer indicates the company that produced the tag electronics, duty cycle indicates that number of hours the tag was turned on and available to transmit or off and conserving battery power, the repetition rate indicates how often the tag attempted an uplink to the Argos satellite system and the maximum number of transmissions indicates the maximum number of transmissions that the tag was programmed to transmit on a daily basis.

PTT	Manufacturer	Duty cycle (hours)	Repetition rate (seconds)	Maximum number of transmissions
53348	Wildlife Computers (delrin stop)	4 on, 8 off, 4 on, 8 off	30	720
53736	Wildlife Computers (delrin stop)	4 on, 8 off, 4 on, 8 off	30	720
64235	Sirtrack	6 on, 18 off	40	unknown
88717	Wildlife Computers (stainless stop)	12 off, 6 on, 6 off	30	720
88718	Wildlife Computers (stainless stop)	12 off, 6 on, 6 off	30	720
88722	Wildlife Computers (stainless stop)	12 off, 6 on, 6 off	30	720
88723	Wildlife Computers (stainless stop)	12 off, 6 on, 6 off	30	720
88725	Wildlife Computers (stainless stop)	12 off, 6 on, 6 off	30	720
88728	Wildlife Computers (stainless stop)	12 off, 6 on, 6 off	30	720
88729	Wildlife Computers (stainless stop)	12 off, 6 on, 6 off	30	720
88732	Wildlife Computers (stainless stop)	12 off, 6 on, 6 off	30	720
88733	Wildlife Computers (stainless stop)	12 off, 6 on, 6 off	30	720
88735	Wildlife Computers (stainless stop)	12 off, 6 on, 6 off	30	720
88738	Wildlife Computers (stainless stop)	12 off, 6 on, 6 off	30	720
88741	Wildlife Computers (stainless stop)	12 off, 6 on, 6 off	30	720

88743	Wildlife Computers (stainless stop)	12 off, 6 on, 6 off	30	720
88744	Wildlife Computers (stainless stop)	12 off, 6 on, 6 off	30	720
88745	Wildlife Computers (stainless stop)	12 off, 6 on, 6 off	30	720
88746	Wildlife Computers (stainless stop)	12 off, 6 on, 6 off	30	720
96385	Wildlife Computers (stainless stop)	12 off, 6 on, 6 off	30	720
96386	Wildlife Computers (stainless stop)	12 off, 6 on, 6 off	30	720
96390	Wildlife Computers (stainless stop)	12 off, 6 on, 6 off	30	720
96398	Wildlife Computers (stainless stop)	12 off, 6 on, 6 off	30	720
96403	Wildlife Computers (stainless stop)	12 off, 6 on, 6 off	30	720
96412	Wildlife Computers (stainless stop)	12 off, 6 on, 6 off	30	720
98100	Wildlife Computers (stainless stop)	8 off, 4 on, 8 off, 4 on	30	720
98114	Wildlife Computers (stainless stop)	8 off, 4 on, 8 off, 4 on	30	720
98129	Wildlife Computers (stainless stop)	8 off, 4 on, 8 off, 4 on	30	720
98138	Wildlife Computers (delrin stop)	4 on, 8 off, 4 on, 8 off	30	720
98139	Wildlife Computers (stainless stop)	8 off, 4 on, 8 off, 4 on	30	720

Supplementary S2. Statistical models for behaviour-environment associations.

Table S2.1. Details of environmental variables examined as predictors of whale search behaviour, including data sources, resolution and data citations.

Variable	Description	Data Source	Resolution	Citation
Bathymetric gradient<br><br>(*log bathyg)*	Slope of sea floor	Derived from Smith and Sandwell V13.1 and ETOPO1 bathymetry data. Slope calculated on 0.1-degree gridded depth data. Calculated using the equation given on p. 190 of [105]. Values are log transformed.	0.1 degree	[106]
Seasonal chlorophyll climatology<br><br>(*log ChLa)*	Mean chl-a concentration (mg m^{-3}) calculated across a 3 month season, where season1/Spring = Nov – Jan; season 2/Summer = Feb –Apr.	Surface chlorophyll-a concentration (mg m^{3}) derived from satellite ocean colour. Sourced from the monthly SeaWiFS 9 km resolution product over the period 2001-2010, with the improved Southern Ocean-specific algorithm applied[107]. Values are log transformed.	9 km	[108]
Distance to ice<br><br>(*sqrt dist ice*)	Great circle distance (km) to the 15% ice concentration contour	Derived from the daily NSIDC SMMR-SSM/I sea ice concentration data. Values are square root transformed to highlight the region close to ice edge.	25 km	[109]
Ice melt rate<br><br>(*melt rate*)	Spatial gradient in the rate of ice retreat.	For each season a spatial raster was created of the day of ice melt (i.e., the day of season any pixel was last covered by ice of at least 15% concentration). Data were derived from the daily NSIDC SMMR-SSM/I sea ice concentration data and reprojected to a 0.23° longitude-latitude grid. To capture negative gradients (e.g. early melt close to the Antarctic continent in the south) a first difference calculation	25km	[109]

		was applied north-to-south. Values are square root transformed (with sign retained).		
Ice cv lag 2<br><br>(*ice cv lag 2)*	Coefficient of variation (CV) in the sea ice concentration two months prior	We used the coefficient of variation (CV = σ/μ), i.e. the ratio of the standard deviation to the mean, as an indicator of the ice edge variability. Where there is generally low mean ice concentration and high variance there will be a high CV giving a spatial representation of the marginal ice zone. The CV for each month was calculated from AMSR-E daily sea ice concentration at 6.25km resolution. So for example a tracking location in March would be associated with a CV (lag 2) value for that spatial pixel from January.	6.25 km	[110]
Ice mean lag 1<br><br>(*ice mn lag 1*)	Mean sea ice concentration (%) one month prior	Mean ice concentration (%) for the month prior. Calculated from AMSR-E daily sea ice concentration at 6.25km resolution	6.25km	[110]

Table S2.2. Exploratory model series examining environmental predictors of whale movement behaviour (n = 1442). The discrete behavioural state estimate (*b*, where 1 = 'transit', 2 = 'search') is modelled as a binomial response (i.e. the GAMM was fit using a binomial family with a logit link), with individual whale (id) included as a random effect. The GAMM allows for the relationships between behaviour and environmental predictors to be flexible and non-linear. The final GAMM assessed during the resampling procedure is highlighted in bold. All statistical models were fit using the *gamm4* library in R (Wood and Scheipl, 2017).

Formula	GLMM<br><br>LOG LIKELIHOOD	GLMM AIC	Significant parameters at < 0.05 level
b ~ campaign + s(log bathyg) + s(log CHLa) + s(sqrt dist ice) + s(melt rate) + s(ice cv lag 1) + s(ice mn lag 2), random=~(1|id)	-749.9	1531.8	all but *log CHLa* and *ice cv lag 1*
***b ~ campaign + s(log bathyg) + s(log CHLa) + s(sqrt dist ice) + s(melt rate) + s(ice cv lag 2) + s(ice mn lag 1)** , random=~(1|id)*	**-749.4**	**1530.8**	**all but *log CHLa***
b ~ campaign + s(log bathyg) + s(sqrt dist ice) + s(melt rate) + s(ice cv lag 2) + s(ice mn lag 1) , random=~(1|id)	-751.2	1530.3	all
b ~ campaign + s(log bathyg) + s(sqrt dist ice) + s(melt rate) + s(ice cv lag 1) + s(ice mn lag 2) , random=~(1|id)	-751.6	1531.2	all but *ice cv lag 1*
b ~ campaign + s(log bathyg) + s(sqrt dist ice) + s(ice cv lag 2) + s(ice mn lag 1) , random=~(1|id)	-758.4	1540.8	all
b ~ campaign + s(sqrt dist ice) + s(ice cv lag 2) , random=~(1|id)	-765.7	1547.5	all

Initially the variables describing mean ice concentration at both one and two months prior (*ice mn lag 1, ice mn lag 2*) as well as the CV of ice concentration one and two months prior (*ice cv lag 1, ice cv lag 2)* were included in exploratory analyses. The one and two month terms were highly correlated ($r > 0.8$)

therefore only one term describing ice concentration lag and one term describing ice variability lag could be included in the final model. The AIC values guided model selection, although the *log CHLa* (ΔAIC +0.5) was retained in order to include at least one predictor representing biological productivity during the resampling procedure.

Table S2.3. Full results for the environmental GAMM. The discrete behavioural state estimate (*b*, where 1 = ‘transit’, 2 = ‘search’; n = 1442) from the hierarchical state-space switching model is modelled as a binomial response (i.e. the GAMM was fit using a binomial family with a logit link), with individual whale (id) included as a random effect (S.D. estimate: 1.681). The model formula is:

b ~ campaign + s(log bathyg) + s (log CHLa) + s(sqrt dist ice) + s(melt rate) + s(ice cv lag 2) + s(ice mn lag 1), random=~(1|id)

Parametric coefficients:

Campaign	Estimate	Stdandard error	z value	Pr(>|z|)
(Intercept) AU-Eden	0.971	0.6185	1.57	0.116
ANT	-0.6066	0.8482	-0.715	0.474
AU-SC	-0.4002	1.2028	-0.333	0.739

Approximate significance of smooth terms:

	Estimated degrees of freedom	Reference degrees of freedom	Chi square statistic	*p*-value
s(log bathyg)	1	1	7.27	0.007
s (log CHLa)	1	1	2.49	0.114
s(sqrt dist ice)	8.27	8.27	56.71	<0.001
s(melt rate)	1	1	7.06	0.008
s(ice cv lag 2)	3.8	3.8	26.45	<0.001
s(ice mn lag 1)	2.41	2.41	7.25	0.026

S2.4. Examining for effects relating to spatial and temporal autocorrelation.

Spatial. Spatially autocorrelated data can affect the smoothness estimation of model terms; i.e., the estimation procedure in GAM is likely to under-smooth. To further examine for effects relating to spatial autocorrelation, we therefore also implemented the same model including a spatial term interaction. Spatial coordinates (i.e. longitude and latitude) were transformed prior using a Lambert equal area transformation, and included in the model as explanatory variables via a full tensor product smooth on x and y (i.e., t2(x,y)). In a second implementation these were included as separate smooth terms (i.e., s(x) and s(y)).

In both cases the GAMMs retained the significance of the ice variables representing distance to the ice edge and ice melt rate; despite, as to be expected, spatial smooths absorbing much of the variation. There was some exchange in term significance between the lagged ice variables such that the mean ice concentration prior was prioritised rather than the ice variability in both spatial models. This indicates that the influence of the ice variability term likely mimics/is mimicked by the spatial term; i.e., it is likely to be associated with the focussed spatial area 145 -175°E around the Balleny Islands (66°55’S 163°45’E) where the majority of foraging behaviour was identified. Overall, these results reinforce the prominent role that features of the marginal ice zone play in influencing the search behaviour of humpback whales.

i) Full tensor product smooth on x and y (t2(x,y)

b ~ campaign + t2(x, y) + s(log_slope) + s(log_chl_clim) + s(sqrt_dist_ice) + s(melt_rate_zero) + s(ice_cv_lag2) + s(ice_mn_lag1), random=~(1|id)

Approximate significance of smooth terms:

	Estimated degrees of freedom	Reference degrees of freedom	Chi square statistic	*p*-value
t2(x,y)	17.770	17.770	101.456	<0.001
s(log bathyg)	1	1	1.82	0.178
s (log CHLa)	1	1	1.82	0.178
s(sqrt dist ice)	7.760	7.760	58.16	<0.001
s(melt rate)	1	1	5.02	0.025
s(ice cv lag 2)	1.001	1.001	0.54	0.46
s(ice mn lag 1)	3.41	3.41	19.76	<0.001

ii) Separate smooths on terms x and y (s(x) and s(y))

b ~ campaign + s(x) + s(y) + s(log_slope) + s(log_chl_clim) + s(sqrt_dist_ice) + s(melt_rate_zero) + s(ice_cv_lag2) + s(ice_mn_lag1) , random=~(1|id)

Approximate significance of smooth terms:

	Estimated degrees of freedom	Reference degrees of freedom	Chi square statistic	*p*-value
s(x)	8.020	8.020	89.977	<0.001
s(y)	7.917	7.917	134.449	<0.001
s(log bathyg)	1	1	0.003	0.956
s (log CHLa)	1	1	5.901	0.015
s(sqrt dist ice)	8.034	8.034	60.189	<0.001
s(melt rate)	1	1	4.787	0.029
s(ice cv lag 2)	1	1	0.482	0.488
s(ice mn lag 1)	3.110	3.110	20.858	<0.001

Temporal. To further examine for effects related to temporal autocorrelation we successively thinned the full tracking dataset, that is we employed destructive sampling. We performed experiments deleting every 4th (removes 25%), 3rd (removes 33%) and 2nd (removes 50%) data row per track and retaining every 3rd (removes 66%) and 4th (removes 75%) data row per track.

As to be expected, with more destructive resampling comes a loss of information and a degradation of the strength of the signal across all environmental variables. Even so, the ice related variables continue to be the most commonly retained across models. As such, the environmental interpretation that inclusion of the ice-related variables was the most highly resilient to uncertainty, appears robust.

Subsampling results. *P*-values are given for the original GAMM fit to the most probable discrete behavioural state estimate from the hSSSM, and indicate the approximate significance of GAMM smooth terms based on Chi-sq. statistics. Second column (N sign. /100) indicates the number of times each environmental variable was determined to be significant (based on *p*-value < 0.05) under the resampling procedure (n = 100 iterations), which resamples randomly from the posterior the estimates for both behavioural state and location.

	Remove every 4th sample (delete 25%)		Remove every 3rd sample (delete 33%)		Remove every 2nd sample (delete 50%)		Retain every 3rd sample (delete 66%)		Retain every 4th sample (delete 75%)	
	p-value	N sign. /100	*p*-value	N sign. /100	*p*-value	N sign. /100	*p*-value	N sign. /100	*p*-value	N sign. /100
log bathyg	0.052	10	0.008	15	0.148	8	0.677	0	0.049	16
log CHLa	0.043	4	0.215	2	0.093	7	0.331	1	0.609	7
sqrt dist ice	<0.001	53	<0.001	22	0.001	34	<0.001	52	0.496	24
melt rate	0.023	51	0.036	36	0.200	11	0.091	35	0.072	17
ice cv lag2	0.001	81	0.024	59	0.040	60	0.010	55	0.050	43
ice mn lag1	0.826	16	0.102	17	0.739	6	0.987	8	0.011	22

Supplementary S3. Animation depicting each monthly coefficient of variation (CV) in ice concentration.

The top panel is monthly mean ice concentration, the middle panel is variance in monthly ice concentration and the bottom panel is monthly CV in ice concentration. Higher ice concentration, variance and CV are shown as warmer colours as can be seen in the scale bar in the right hand side of each panel. The date for all three animated panels is given above the top panel in the format of 'yyyy-mm-dd.'

State space modelled track location estimates are mapped on the monthly CV in ice concentration panel (bottom). Location estimates with no time lag are represented as white points, location estimates at a one month time lag are represented as pink points and location estimates at a two month time lag are represented as magenta points. For example, all white points on the plot of December 2008 ice concentration CV ('2008-12-01') are all location estimates generated in December 2008. All pink points represent location estimates generated one month prior (November 2008). Magenta points represent location estimates generated two months prior (October 2008) which is the *ice cv lag 2* variable included in the final full GAMM.
